# A study of the temporal robustness of the growing global container-shipping network

**DOI:** 10.1038/srep34217

**Published:** 2016-10-07

**Authors:** Nuo Wang, Nuan Wu, Ling-ling Dong, Hua-kun Yan, Di Wu

**Affiliations:** 1 Department of Transportation Management, Dalian Maritime University, Dalian 116026, China; 2 Sino-US Global Logistics Institute, Shanghai Jiao Tong University, Shanghai 200030, China

## Abstract

Whether they thrive as they grow must be determined for all constantly expanding networks. However, few studies have focused on this important network feature or the development of quantitative analytical methods. Given the formation and growth of the global container-shipping network, we proposed the concept of network temporal robustness and quantitative method. As an example, we collected container liner companies’ data at two time points (2004 and 2014) and built a shipping network with ports as nodes and routes as links. We thus obtained a quantitative value of the temporal robustness. The temporal robustness is a significant network property because, for the first time, we can clearly recognize that the shipping network has become more vulnerable to damage over the last decade: When the node failure scale reached 50% of the entire network, the temporal robustness was approximately −0.51% for random errors and −12.63% for intentional attacks. The proposed concept and analytical method described in this paper are significant for other network studies.

In recent years, complex networks have been used to represent natural and man-made systems, such as genetics, proteomics and metabolomics^1^, the study of neurological diseases^2^, the World Wide Web^3^ and transportation networks^4^. Network failure has recently been a research focus in complex network theory^5^,^6^,^7^ and has attracted many scholars’ attention^4^,^8^,^9^,^10^,^11^. Several researchers have addressed the basic characteristics and metrics of complex networks in terms of the probability distribution of node degrees and network connectivity^12^,^13^,^14^,^15^, the quantitative assessment of network robustness and vulnerability^16^,^17^,^18^, a network’s recovery ability and strategy after partial failure^6^,^19^,^20^, the improvement of network survivability^15^,^21^,^22^,^23^, and the evaluation of network efficiency and simulation-based analytical methods^24^,^25^,^26^. Studies of transportation networks have mainly focused on highway networks^27^,^28^,^29^ and have included planning the scale of an efficient transportation network^30^,^31^,^32^; the effect of different travel choice dimensions on a network’s vulnerability using the traffic demand combination model^17^,^33^,^34^,^35^; and analysis of transportation networks from the perspectives of complexity, geographic spatial structure, organization and efficiency, and open systems^36^,^37^,^38^,^39^.

Although many studies have addressed the characteristics of transportation networks using complex network theory, some problems still require further work. The main problems include the following: (i) When analysing network node failures, they mainly investigate the network’s metrics and seldom study changes that occur before or after a node is removed. (ii) They rarely carry out comparative study of the change extent of a network’s metrics at different points in time. (iii) They lack a quantitative analytical method for objectively judging the extent of change in a network’s stability. We do not believe it is sufficient to study only a static network at a single point in time. Indeed, to observe an important objective-property of a network as it grows, we establish a new concept of network’s temporal robustness and a general analytical method. And it is vitally important to clearly monitor a network’s growth behaviour.

Temporal robustness is defined as the trend in a network’s ability to overcome external interferences and maintain its original function during the growth process. In other words, if robustness is based on a single time point, temporal robustness is the trend of the changes in a network’s stability over a certain time period. If a network’s robustness increases over time, then its temporal robustness also increases and vice versa.

The container-shipping network, which operates along fixed routes, was developed over the last 50 years and has rapidly become a new type of transportation system. Because the global economy relies heavily on the container-shipping network, when unexpected events (e.g., earthquakes, tsunamis, dock worker strikes and terrorist attacks) occur, the shipping network fails locally, leading to fluctuations in the world economy^40^,^41^. Therefore, studying the temporal robustness of the global container shipping network as it expands is of vital importance for establishing a security mechanism for the world economy and for port planning and route design.

Based on the proposed problem and the actual demand, we introduce the concept of network temporal robustness and a quantitative analytical method. Using the main global container-shipping companies’ data on calling ports and route distributions from 2004 and 2014, we quantitatively judge the changes in the temporal robustness of the global container-shipping network. The effectiveness of the analytical method described here is also demonstrated.

## Results

### Network model

Unlike traditional sea-based bulk cargo transportation, container shipping is a complex network system comprising pivotal ports, main ports, spoke ports and feeder ports, in that order^42^,^43^,^44^. The pivotal ports and main ports are responsible for the container-transhipment business, and each liner is anchored by dense routes. In contrast, the spoke ports and feeder ports mainly link with nearby pivotal ports, and each liner is anchored to a round trip with sparse routes^45^,^46^,^47^. We selected statistics for all calling ports and routes (excluding repeated routes) operated by the top 25 shipping companies (constituting more than 80% of the global transportation capacity) at two time points (2004 and 2014). In total, there were 503 container ports and 1436 routes in the global container-shipping network in 2004. These values increased to 634 (26% increase) and 2728 (90% increase), respectively, in 2014. We observed that the global container-shipping network expanded rapidly during the last decade.

For the theoretical analysis, all the ports in the global container-shipping network are abstracted to nodes; *Z* is the number of ports, and *V* = {*v*_1_, *v*_2_, *v*_3_, …, *v*_*Z*_} is the set of ports. The connectivity between *v*_*i*_ and *v*_*j*_ is abstracted to a network link. If *v*_*i*_ connects with *v*_*j*_, then *e*_*i,j*_ = 1; otherwise, *e*_*i,j*_ = 0. The adjacency matrix (*E*_*Z*×*Z*_) is defined as follows:


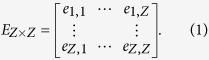


**Figure 1:**
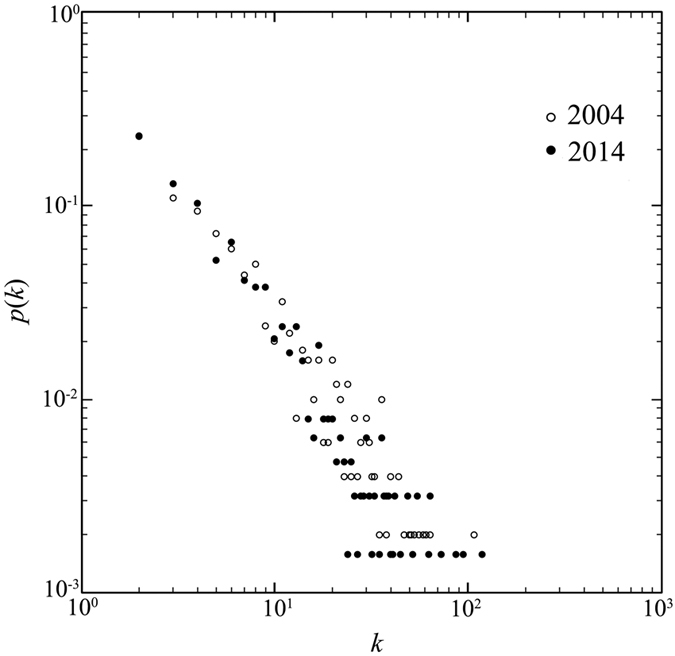
The distribution of ports’ degrees in the container-shipping network shown on a log-log plot.

In accordance with the proposed method, we represent the global container-shipping network as an undirected and unweighted graph *W *= (*V*, *E*). We graph the probabilities of all the node degrees on a log-log plot to obtain the distribution (Fig. 1). It is easily proven that the connectivity distribution *p*(*k*) without *k* = 1 is as follows:


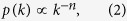


where *k* is the degree of the node, *k* ∈ {*k*_1_, *k*_2_, …, *k*_*Z*_}, *k*_*i*_ is the degree of node *v*_*i*_, and *n* is the power-law exponent.

Using fitting^48^, we obtain *n*_2004_ = 1.6843 and *n*_2014_ = 1.6549, indicating that *p*(*k*) has a power law distribution, and the container-shipping network can be characterized as a scale-free network. A few highly connected nodes have large numbers of links, but most nodes have one or two links. It shows many spoke ports with few lines have connected to the container network during 2004 to 2014 in reality.

### Node-removal strategies

Ports in the shipping network can fail for a variety of reasons, most of which belong to two major classes. One class contains objective factors, such as typhoons, earthquakes and tsunamis, which can occur at any port. We call members of this class random errors. In this paper, we use randomly generated port sequence data as the removal strategy to simulate this failure. The other class contains man-made attacks, such as terrorist attacks, which we call intentional attacks. We suppose that a more important port is more likely to be attacked, and we remove the port with the largest degree to simulate this type of failure. These two failure strategies cause the network to react differently, and we investigated these differences.

### Calculation procedure

To quantitatively analyse the network’s temporal robustness, we select four metrics that are closely related to the shipping network: the network’s average degree (<*k*>), the network’s clustering coefficient (*C*), the proportion of isolated nodes in the network (*N*) and the network’s average shortest-path length (*L*). Detailed descriptions and calculations of all the metrics are provided in the Methods section. The procedure to calculate the network’s temporal robustness under a designated failure strategy is as follows:

1. Based on the container-shipping network data from different years, we calculate the extent of change of the network metrics before and after gradual node removal under the designated failure strategies and obtain Δ*K,* Δ*C,* Δ*N* and Δ*L* at different points in time (details are provided in the Analysing the network’s metrics subsection).
2. Pressure test. Based on the extent of change of each metric, we determine the proportion of node removal causing network failure (*H*_*c*_), thus get the half-failure degree (*G*_*s*_) at different points in time. Then, we determine the sensitivity coefficient (*O*_*s*_) of each metric based on the ratio of the half-failure degrees in different years. Using the proportion of each sensitivity coefficient, we obtain the weight (*Q*_*s*_) that each metric contributes to the network’s temporal robustness (details are provided in the Pressure test subsection).
3. Using the stated failure scale, we calculate the value ( 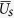 ) that each metric contributes to the network’s temporal robustness (details are provided in the Failure scale subsection).
4. We obtain a quantitative value of the network’s temporal robustness under the designated failure strategy (*F*) by multiplying each contribution ( 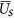 ) with its weight (*Q*_*s*_) (details are provided in the Quantitative value subsection).
5. End.

**Figure 2:**
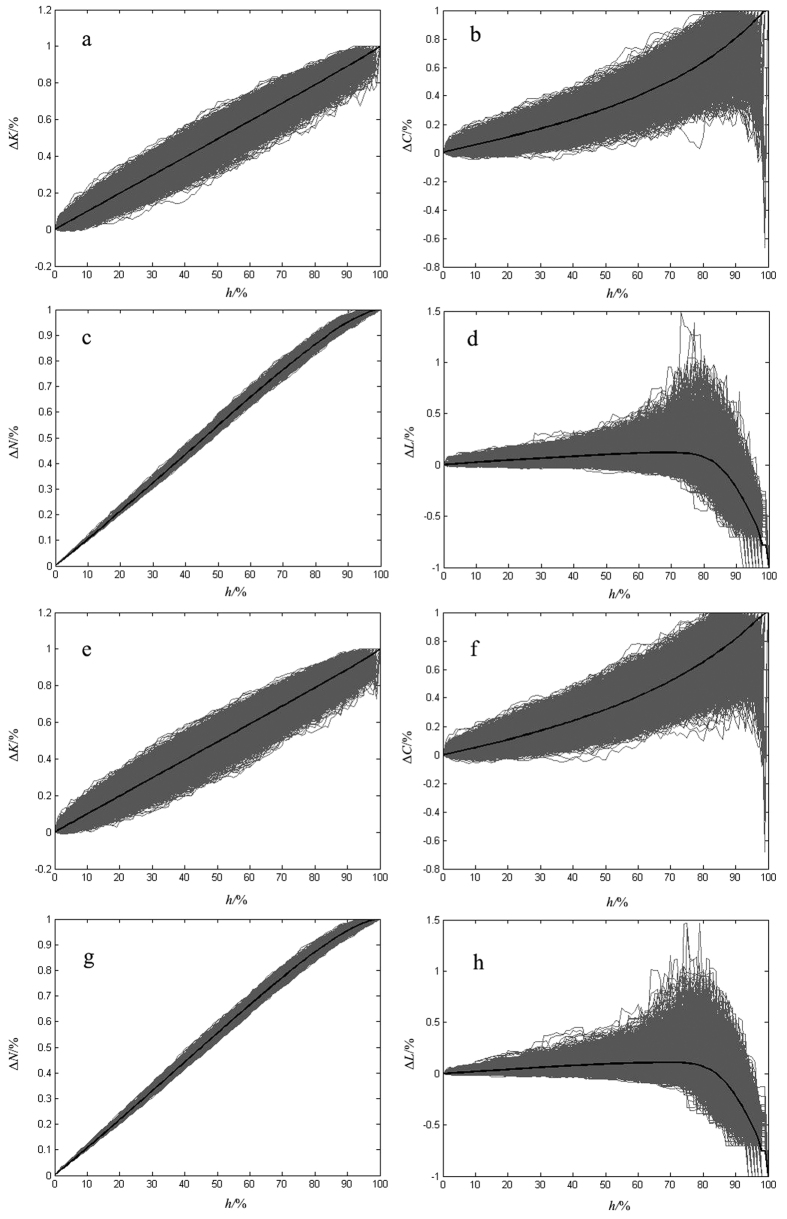
The results of 10000 simulations for random errors. (**a**–**d**) are simulation results in 2004, (**e**–**h**) are simulation results in 2014. (**a**,**e**) show the change of network’s average degree, and Δ*K* is the extent of change while *h* means the proportion of node removed. (**b**,**f**) show the change of network clustering coefficient, and Δ*C* is the extent of change. (**c**,**g**) show the change of network’s proportion of isolated nodes, and Δ*N* is the extent of change. (**d**,**h**) show the change of network’s average shortest-path length, and Δ*L* is the extent of change. The black line in every panel represents the mean value of 10000 simulations.

**Figure 3:**
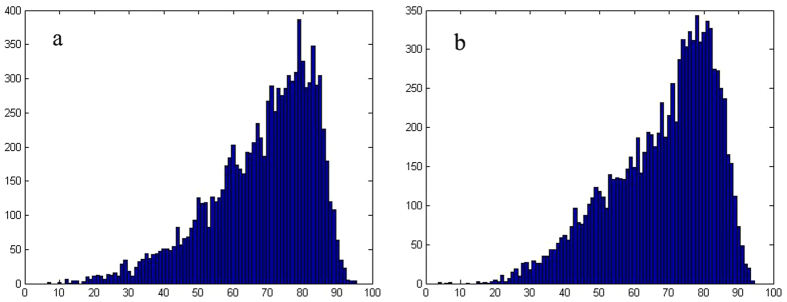
The histogram of network failure times under different proportions of node removal for random errors. (**a**) shows the result in 2004 while (**b**) shows the result in 2014.

**Figure 4:**
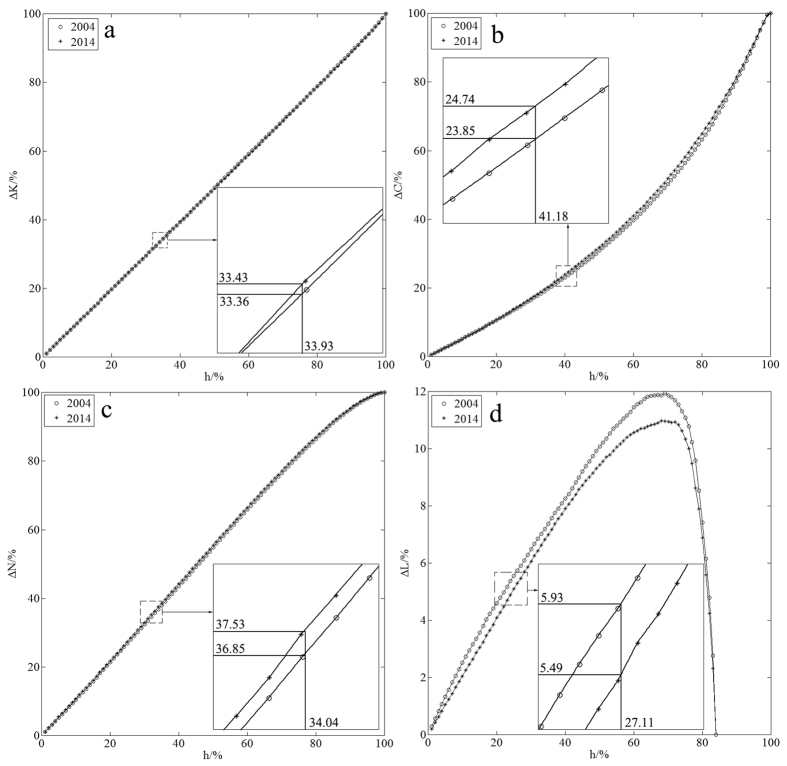
Pressure test analysis diagrams for random errors. (**a**) shows the change of network’s average degree, (**b**) shows the change of network clustering coefficient, (**c**) shows the change of network’s proportion of isolated nodes, and (**d**) shows the change of network’s average shortest-path length.

**Figure 5:**
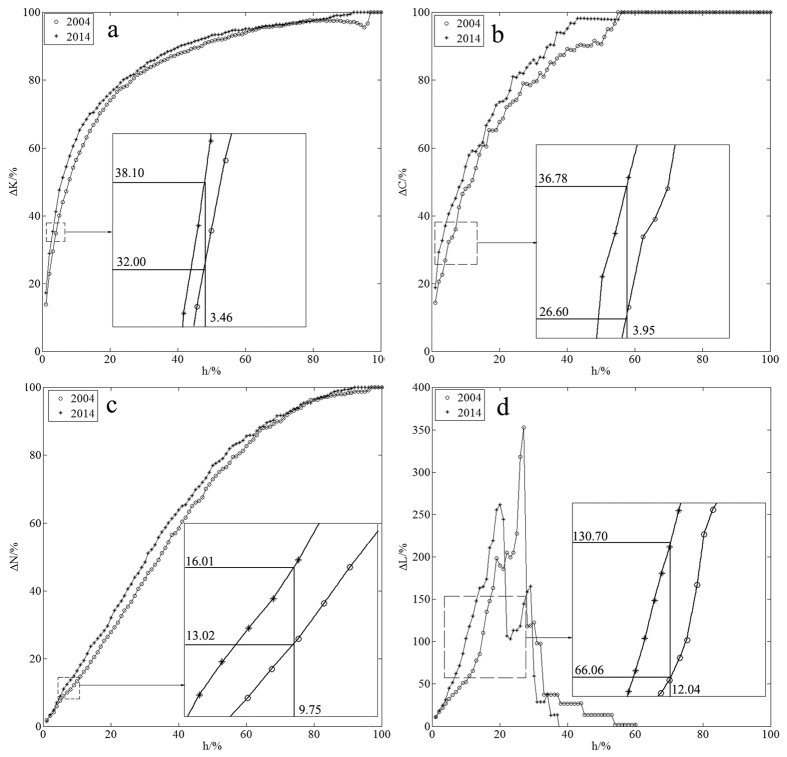
Pressure test analysis diagrams for intentional attacks. (**a**) shows the change of network’s average degree, (**b**) shows the change of network clustering coefficient, (**c**) shows the change of network’s proportion of isolated nodes, and (**d**) shows the change of network’s average shortest-path length.

Analysing the extent of change of all the metrics, we found the network’s average shortest-path length was the first metric to fail. Based on this, we determined the proportion of node removal causing network failure. To monitor a network’s growth behaviour for random errors, we took 10000 random simulations and used the mean value of these simulations as the basic data (see Fig. 2). Through the statistical analysis on the proportion of node removal causing network failure for random errors (see Fig. 3), we got the proportions 69% in 2004 and 68% in 2014, while the standard deviations were 0.1493 and 0.1503, respectively. Also we got the proportions of node removal causing network failure for intentional attacks 27% in 2004 and 20% in 2014. We computed these values using the calculations found in the Methods section. Using the calculation procedure above, we easily obtained the result of network temporal robustness. The results show the contributions of the network’s average degree, the network’s clustering coefficient, the proportion of isolated nodes and the average shortest-path length to network temporal robustness are −0.02%, −0.22%, −0.17% and −0.10%, respectively, for random errors (see Fig. 4), while the contributions are −1.65%, −2.04%, −0.81% and −8.13%, respectively, for intentional attacks (see Fig. 5). We accumulated all the contributions and determined that the container-shipping network’s temporal robustness was approximately −0.51% for random errors and −12.63% for intentional attacks during 2004 and 2014 (Table 1). The results show the network temporal robustness decreased in both cases, and the decrease in the intentional attack case was more drastic.

## Discussion

We draw the following general conclusions:

- The statistical data show that the port connectivity has a power law distribution and that the global container-shipping network belongs to a class of scale-free networks. The global container-shipping network expanded rapidly form 2004 to 2014 and it was a growing complex network.
- To explore the trend in a network’s ability to overcome external interferences and maintain its original function during the growth process, we proposed the concept of network temporal robustness. We performed a network pressure test to analyse the change trend of the network’s robustness at two time points (2004 and 2014). The proposed perspective and method are very important to achieving a thorough understanding of the network system.
- By analysing changes in the network’s metrics at two time points (2004 and 2014), we determined the weights and values of the metrics that affect the network when it is subjected to both random errors and intentional attacks. We found that the temporal robustness of the global container-shipping network was −0.51% for random errors and −12.63% for intentional attacks during 2004 and 2014, when failure scale approached the network’s half-failure point. The results show temporal robustness decreased in both cases, and the decrease for intentional attacks was more drastic.

The above analysis shows that the temporal robustness of the global container-shipping network develops in a negative direction currently. The rapid growth of container transportation in recent years promotes the nearby ports located in the main channel to form a dual-core hub layout^49^. Many important pivotal ports develop in concomitance with a nearby pivotal port, such as Singapore and Port Klang, Shanghai and Ningbo, Busan and Yokohama, Hong Kong and Shenzhen, Los Angeles and Long Beach. These highly connected ports actually play a backup mechanism in the network. Once a pivotal port fails for intentional attacks, the nearby pivotal port will immediately undertake the transportation business. This mechanism can reduce the influence to the container-shipping network. Further research is about the temporal robustness of network self-adjust mechanism.

## Methods

### Analysing the network’s metrics

On the basis of the complex network theory^3^,^5^,^50^ and the studies of robustness^10^,^13^,^51^, several metrics can be selected to describe the global feature of complex network. Through the definitions of the metrics, we found some metrics had correlation with each other, such as network average degree and density, average shortest-path length and efficiency, so we selected only one representative metric in each group to avoid duplication. Considering the characteristics of the container-shipping network^41^,^52^,^53^,^54^, we finally selected four distinct and representative metrics: the network’s average degree, the network’s clustering coefficient, the network’s proportion of isolated nodes and the network’s average shortest-path length, to analyse the network’s temporal robustness.

The network’s metrics will change as nodes are gradually removed, and the intensity of this change reflects the network’s ability to resist interference. The change of each metric is analysed as follows.

The node degree *k*_*i*_ is defined as the number of links connecting with node *v*_*i*_. In the global container-shipping network, the value of the degree indicates the importance of one port. If a port is the pivotal or main port, its degree must be larger, whereas the nodes that represent spoke and feeder ports usually have smaller degrees. And the network’s average degree^9^ is denoted by


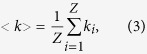


where *<k>* is the network’s average degree and *Z* is the number of ports.

Δ*K* is defined as the extent of change of the network’s average degree before and after node removal, and


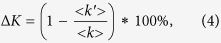


where *<k>* and *<k′>* are the network’s average degrees before and after node removal, respectively.

Using equations (3) and (4), we can obtain the extent of change of the network’s average degree when it is subjected to both random errors and intentional attacks in 2004 and 2014 (Figs 4a and 5a). The results show that the extents of change of the network’s average degree are basically the same development trend and it is a bit larger in 2014 than in 2004. It changes drastically at the beginning for intentional attacks and the extents in both years have exceeded 80% when the proportion of node removal only reaches 27%. It changes slowly in the whole process for random errors and increases simultaneously with the proportion of node removal. The maximal extents of change are 100% in both cases.

The network’s clustering coefficient measures the relationship among neighbouring nodes in the network and reflects the network’s degree of aggregation. If *v*_*i*_ connects directly with many other ports, these ports are its neighbours. The local clustering coefficient (*C*_*i*_)^7^ is defined as follows:


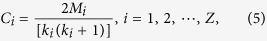


where *M*_*i*_ is the number of links connecting *v*_*i*_ with its neighbours.

The network’s clustering coefficient (*C*) is the average of all the local clustering coefficients; therefore,


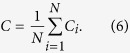


Obviously, 0 ≤ *C* ≤ 1, and all nodes are isolated when *C* = 0. The network becomes a complete graph when *C* = 1, implying that any two nodes in the network are connected. To determine the pivotal characteristic, Δ*C* is defined as the extent of change of the network’s clustering coefficient before and after node removal, and


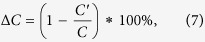


where *C* and *C′* are the network’s clustering coefficient before and after node removal, respectively.

Using equations (6) and (7), we can obtain the extent of change of the network’s clustering coefficient for both random errors and intentional attacks in 2004 and 2014 (Figs 4b and 5b). The results show that the trend of the extent is basically the same in the two years for random errors, and the extent of change in 2014 is a bit greater than that in 2004. It changes drastically for intentional attacks, and the maximum difference of the two years can reach 10%. But the maximal extents of change of network’s clustering coefficient are 100% in both cases.

Some isolated nodes remain after the nodes are removed because of the different strategies. The proportion of isolated nodes reflects the degree of dispersion, and we define Δ*N* as the extent of change of the network’s proportion of isolated nodes before and after node removal, and


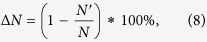


where *N* and *N′* are the network’s proportion of isolated nodes before and after node removal, respectively.

Using equation (8), we can obtain the extent of change of the network’s proportion of isolated nodes for both random errors and intentional attacks in 2004 and 2014 (Figs 4c and 5c). The results show that the extent of change of the network’s proportion of isolated nodes is a bit greater in 2014 than in 2004. The extents change slowly, and the maximal extents are 100% for both random errors and intentional attacks.

A network’s complexity can be characterized using its average shortest-path length (*L*), which is defined as the average of the shortest paths between every pair of nodes^7^. This value reflects the complexity that one node can reach to another one, and *L* is denoted by


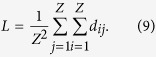


In addition to the distance between two ports, container liner companies comprehensively consider other factors when beginning new routes, including the transportation burden, transhipping costs and transportation conditions. Therefore, we prefer to use the minimum times for transhipment rather than the physical distance between two ports. In other words, the shortest path (*d*_*ij*_) is defined as the smallest number of links that the cargo passed through from *v*_*i*_ to *v*_*j*_. Moreover, the shipping-container network can be considered as an undirected network. Thus, *d*_*ij*_ = *d*_*ji*_ and *d*_*ii*_ = 0. Equation (9) can be simplified as follows:


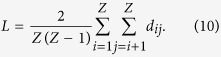


Δ*L* is defined as the extent of change of the network’s average shortest-path length before and after node removal. The network’s average shortest-path length will increase in a certain range as nodes are gradually removed, so we define Δ*L* as follows:


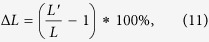


where *L* and *L′* are the network’s average shortest-path lengths before and after node removal, respectively.

Using equations (10) and (11), we can obtain the extent of change of the network’s average shortest-path length for both random errors and intentional attacks in 2004 and 2014 (Figs 4d and 5d). The results show that the maximal extents of change of the network’s average shortest-path length are 11.95% in 2004 and 10.98% in 2014 for random errors. And the maximal extents are 352.62% in 2004 and 261.04% in 2014 for intentional attacks. The network fails much earlier for intentional attacks.

### Quantitative calculation

#### (I) Pressure test

The weight of the network’s metrics is defined as their impact on the network when they change. The key to obtaining a quantitative value for the network’s temporal robustness is to determine the weight. Therefore, we introduce the pressure test method to measure the weight using the following definitions:

1. Metric failure: We call it metric failure if the trend of the change extent is not monotonic with the gradual increasing node removal under the designated failure strategy. And *H*_*c,s*_ is defined as the proportion of node removal when the extent reaches the maximum causing metric failure. If metric *s* is valid all the time, the change trend is monotonic and *H*_*c,s*_= 100%.

2. Network failure: Network failure is determined by the earliest metric failure. *H*_*c*_ is defined as the proportion of node removal which causes network failure, and


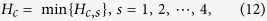


3. Network half-failure degree (*G*_*s*_): Network half-failure degree is defined as the proportion of node removal when the extent of change of metric *s* reaches 50% of its value under network failure.

4. Sensitivity coefficient (*O*_*s*_): The ratio of the half-failure degree of metric *s* at different time points (*T*_1_*<T*_2_) for the designated failure strategy, and


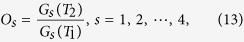


where *G*_*s*_(*T*_1_) and *G*_*s*_(*T*_2_) represent the network half-failure degrees of metric *s* at *T*_*1*_ and *T*_*2*_, respectively.

Weight (*Q*_*s*_): The amount that metric *s* contributes to the network’s temporal robustness for the designated failure strategy, and


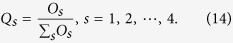


#### (II) Failure scale

Failure scale can be flexibly set to values such as 1%, 5% or 10% of the entire network based on the current demand. However, if we set different percentages, we obtain different values for the temporal robustness. Objectively, the temporal robustness only reflects one network feature and generally provides a useful reference point. Therefore, we should determine the general normalized index.

Based on this concept, we recommend the compromise that takes 50% of network failure as the standard. We take the half-failure degree of network metrics in year *T*_2_ as the proportion of node removed for calculation. Then *U*_*s*_(*T*_*i*_) is defined as the extent of change of metric *s* in year *T*_*i*_ when the proportion of nodes removed reaches *G*_*s*_(*T*_2_), and *R*_*s*_(*T*_*i*_) is defined as the maximal extent of change that metric *s* can reach in year *T*_*i*_. The contribution of metric *s* to the network’s temporal robustness (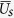) can be obtained as follows:


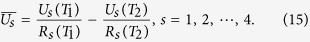


#### (III) Quantitative value

We define *F* as the quantitative value of the network’s temporal robustness and sum the values after multiplying each contribution by its weight. The sum is the quantitative value (*F*),


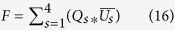


## Additional Information

**How to cite this article**: Wang, N. *et al*. A study of the temporal robustness of the growing global container-shipping network. *Sci. Rep.* **6**, 34217; doi: 10.1038/srep34217 (2016).

## Figures and Tables

**Table 1:** Calculation of the network’s temporal robustness.

Failure strategy	Parameter	Metric				∑
		Average degree	Clustering coefficient	Proportion of isolated nodes	Average shortest-path length	
Random errors	*G*_*s*_(2004) (%)	34.57	42.10	34.83	27.38	──
	*G*_*s*_(2014) (%)	33.93	41.18	34.04	27.11	──
	*O*_*s*_	0.98	0.98	0.98	0.99	3.93
	*Q*_*s*_	0.25	0.25	0.25	0.25	1
	*U*_*s*_(2004) (%)	33.36	23.85	36.85	5.93	──
	*U*_*s*_(2014) (%)	33.43	24.74	37.53	5.49	──
	*R*_*s*_(2004) (%)	100	100	100	11.95	──
	*R*_*s*_(2014) (%)	100	100	100	10.98	──
	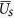 (%)	−0.07	−0.89	−0.68	−0.38	──
	*F* (%)	−0.02	−0.22	-0.17	−0.10	−0.51
Intentional attacks	*G*_*s*_(2004) (%)	5.07	7.55	14.14	18.37	──
	*G*_*s*_(2014) (%)	3.46	3.95	9.75	12.04	──
	*O*_*s*_	0.68	0.52	0.69	0.66	2.55
	*Q*_*s*_	0.27	0.20	0.27	0.26	1
	*U*_*s*_(2004) (%)	32.00	26.60	13.02	66.06	──
	*U*_*s*_(2014) (%)	38.10	36.78	16.01	130.70	──
	*R*_*s*_(2004) (%)	100	100	100	352.62	──
	*R*_*s*_(2014) (%)	100	100	100	261.40	──
	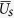 (%)	−6.10	−10.18	−2.99	−31.27	
	*F* (%)	−1.65	−2.04	−0.81	−8.13	−12.63
